# Supplementary material for: Integrating design-of-experiments (DOE) optimization and risk assessment towards a safe and simplified electroporation protocol for Toxoplasma gondii
Source: PLoS Negl Trop Dis. 2026 Apr 8;20(4):e0014194. doi: 10.1371/journal.pntd.0014194 (PMC13086436; doi:10.1371/journal.pntd.0014194)
Supplement: S1 Fig — (PDF) [file pntd.0014194.s001.pdf]

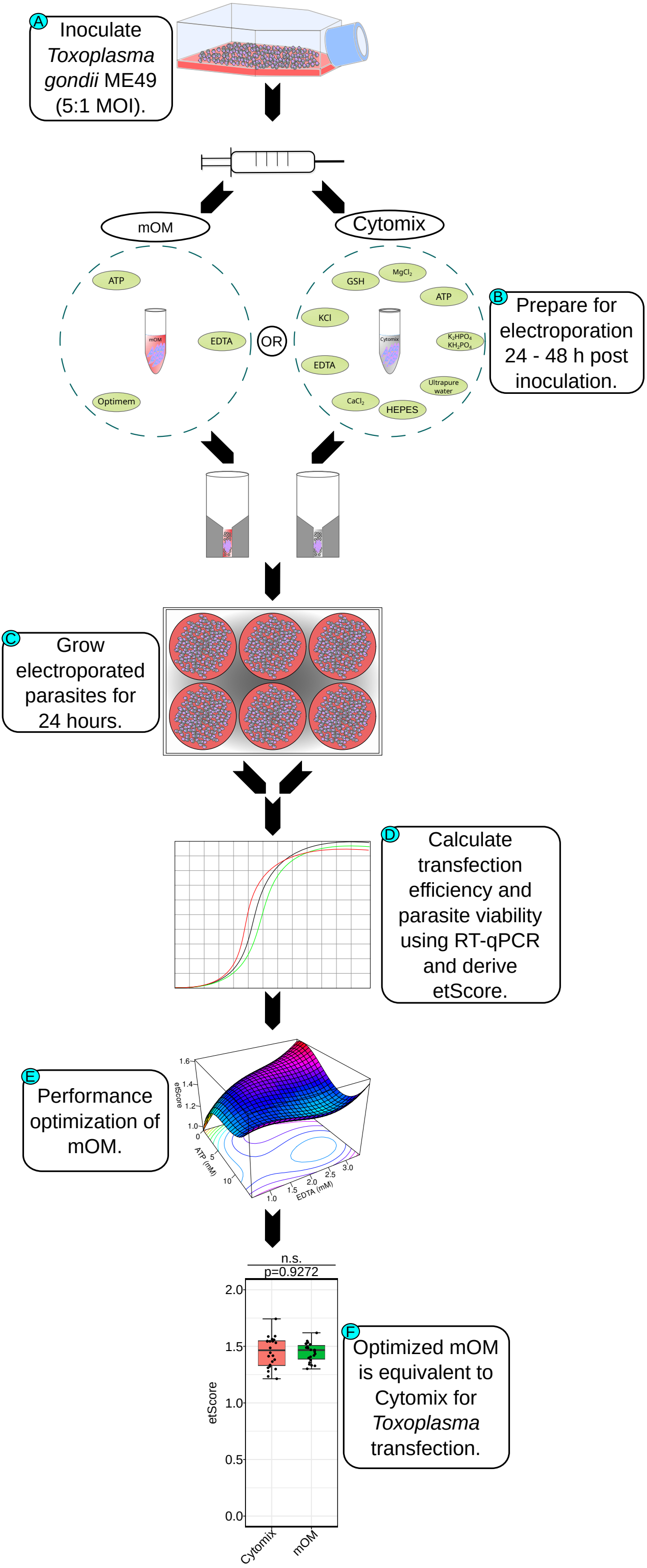

A
Inoculate *Toxoplasma gondii* ME49 (5:1 MOI).
mOM
Cytomix
ATP
EDTA
Optimem
OR
GSH
MgCl2
KCl
K2HPO4
KH2PO4
Ultrapure water
CaCl2
HEPES
B
Prepare for electroporation 24 - 48 h post inoculation.
C
Grow electroporated parasites for 24 hours.
D
Calculate transfection efficiency and parasite viability using RT-qPCR and derive etScore.
E
Performance optimization of mOM.
etScore
1.6
1.4
1.2
1.0
0
5
10
ATP (mM)
1.0
1.5
2.0
2.5
3.0
EDTA (mM)
n.s.
p=0.9272
2.0
1.5
1.0
0.5
0.0
etScore
Cytomix
mOM
F
Optimized mOM is equivalent to Cytomix for *Toxoplasma* transfection.
