# Supplementary figures and images for: Integrating design-of-experiments (DOE) optimization and risk assessment towards a safe and simplified electroporation protocol for Toxoplasma gondii
Source: PLoS Negl Trop Dis. 2026 Apr 8;20(4):e0014194. doi: 10.1371/journal.pntd.0014194 (PMC13086436; doi:10.1371/journal.pntd.0014194)

**ATP**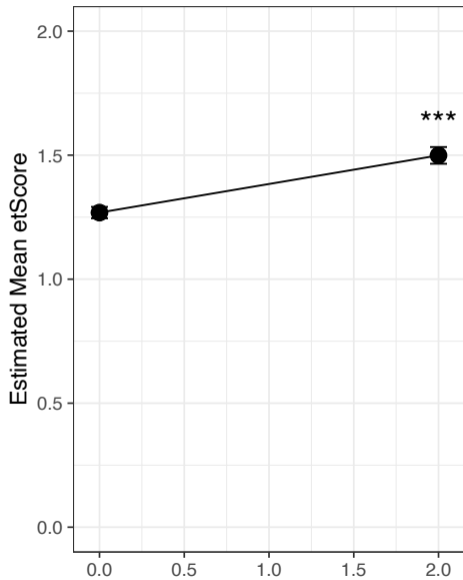**EDTA**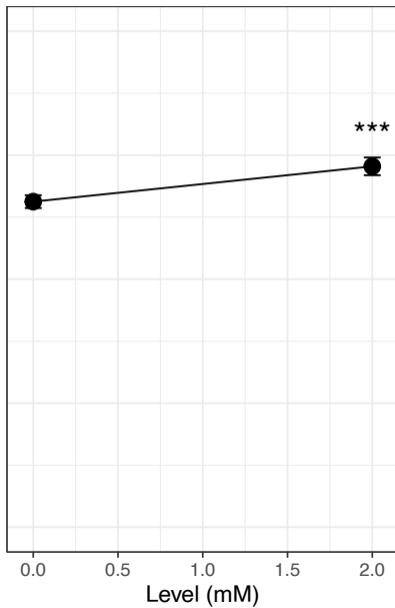**GSH**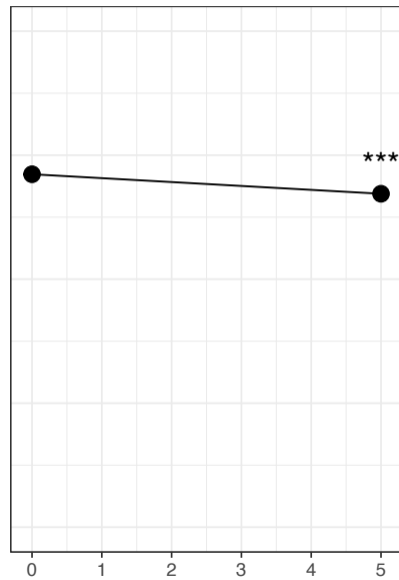

Supplement: S2 Fig — Marginal means are calculated by averaging over the levels of other factors in the model. (PDF) [file pntd.0014194.s002.pdf]

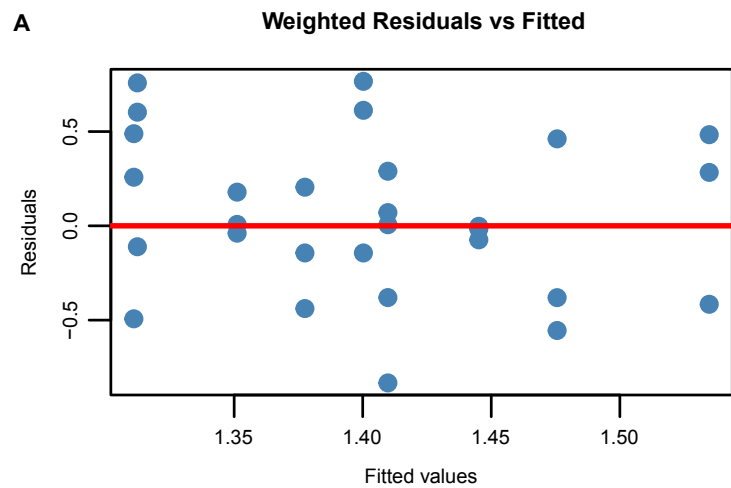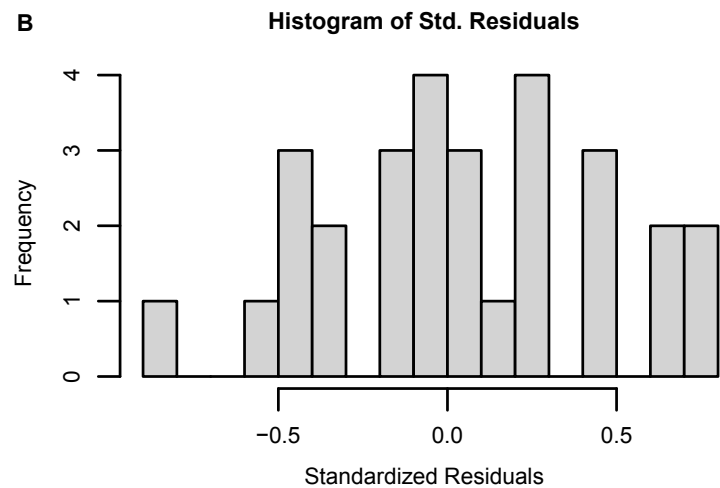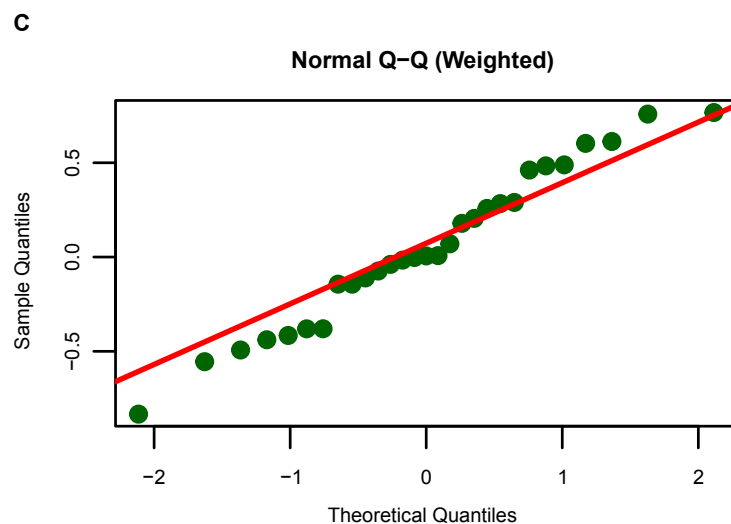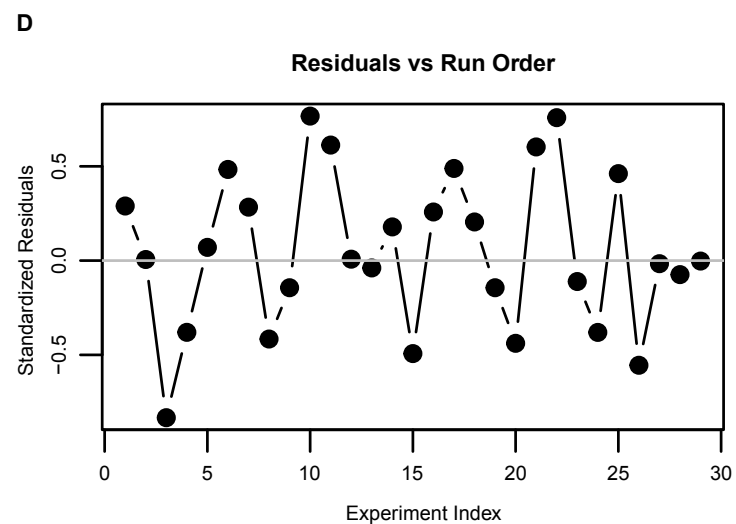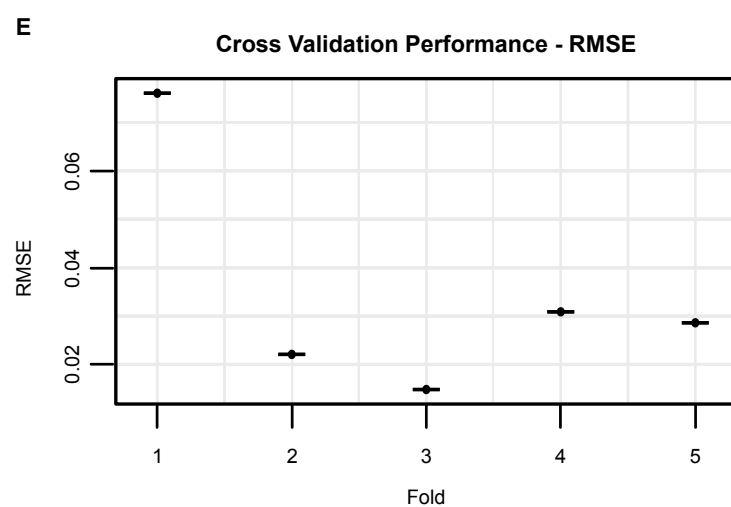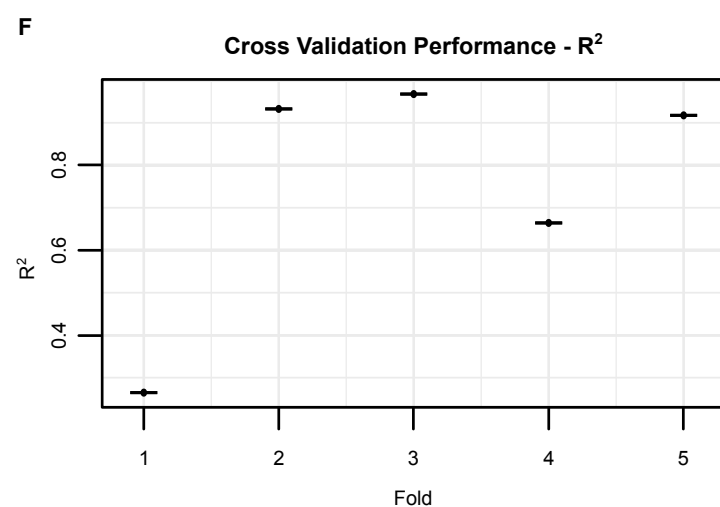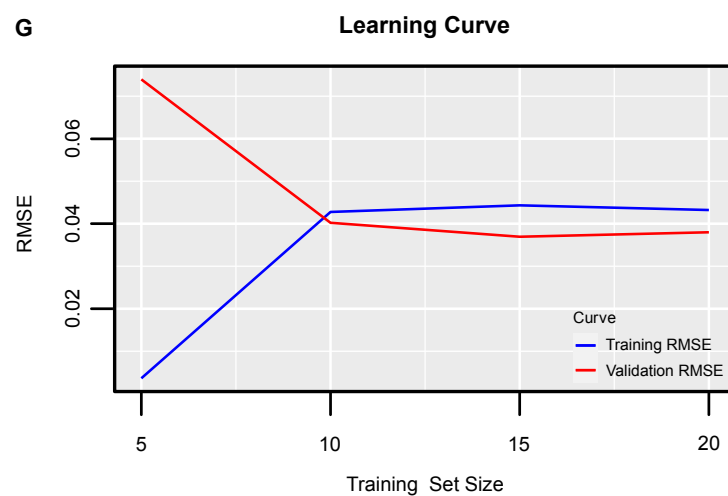

Supplement: S3 Fig — (A) Weighted residuals versus fitted response exhibit a random scatter around zero indicating homoscedasticity. (B-C) Frequency distribution and normal Q-Q (weighted) plots suggest a normal distribution of residuals. (D) Plotting the residuals against the order of observations reveals no discernible trends or patterns. (E-F) Five-fold cross-validation confirms the model’s robustness and predictive power. (G) Learning curves show that the model’s error (RMSE) on both the training and validation datasets converges to similar, low values as more data is incorporated into training. (PDF) [file pntd.0014194.s003.pdf]
