# Supplementary material for: Integrating design-of-experiments (DOE) optimization and risk assessment towards a safe and simplified electroporation protocol for Toxoplasma gondii
Source: PLoS Negl Trop Dis. 2026 Apr 8;20(4):e0014194. doi: 10.1371/journal.pntd.0014194 (PMC13086436; doi:10.1371/journal.pntd.0014194)
Supplement: S4 Fig — Quantification of electroporation outcomes using batch image analysis. (C) Standardized transfection efficiency (percentage of green parasites over red parasites). (D) Standardized viability post electroporation (percentage of red parasites over host cell nuclei, i.e., parasitemia) and (E) etScore calculated as described in the Methods section. p-value derived from Welch’s two sample t test. (DOCX) [file pntd.0014194.s004.docx]

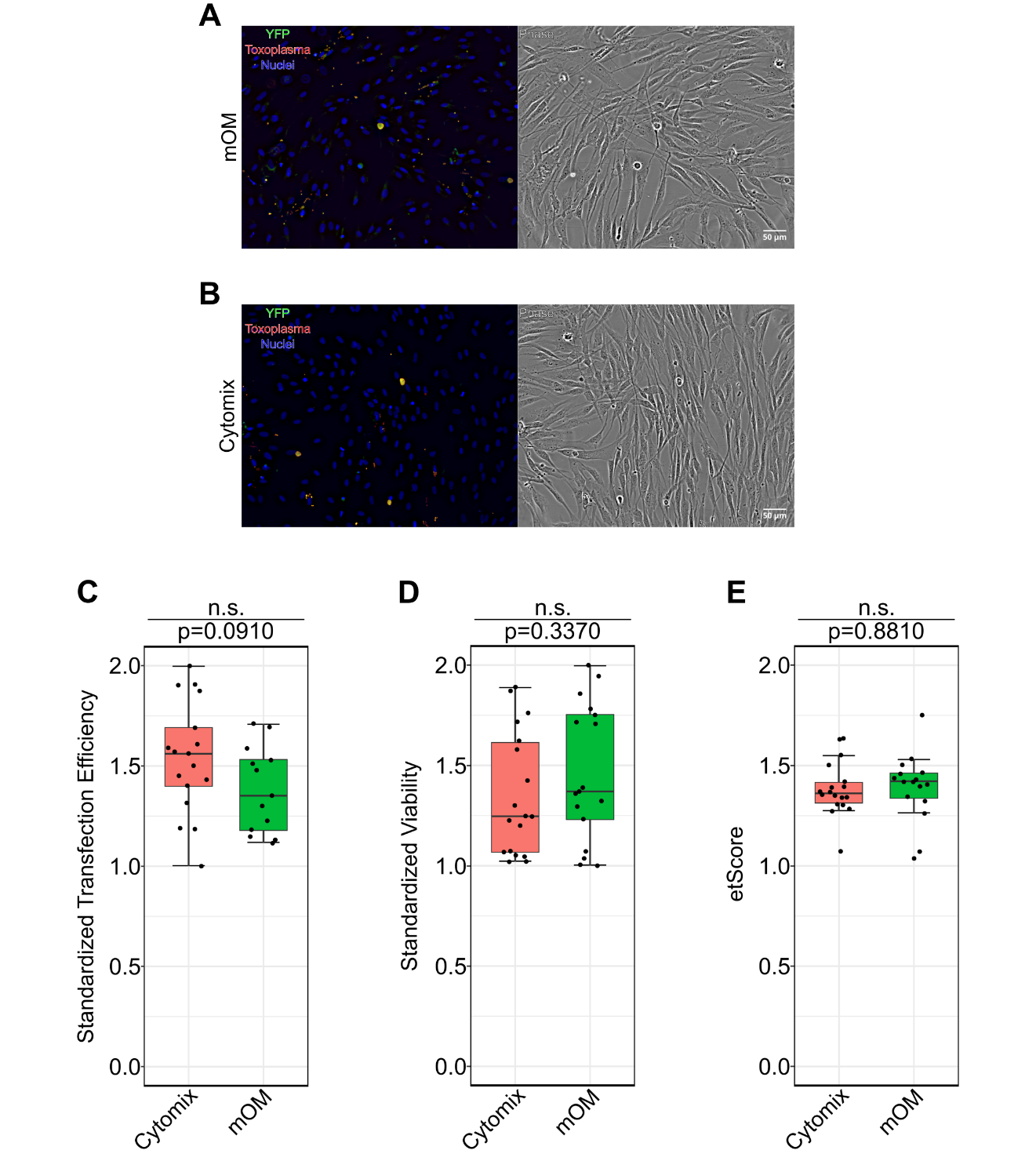
**Fig S4.** Comparison of mOM and cytomix using fluorescence microscopy using **(A)** mOM and **(B)** Cytomix. Quantification of electroporation outcomes using batch image analysis. **(C)** Standardized transfection efficiency (percentage of green parasites over red parasites). **(D)** Standardized viability post electroporation (percentage of red parasites over host cell nuclei i.e. parasitemia) and **(E)** etScore calculated as described in the Methods section. p-value derived from Welch’s two sample t test.
