# Supplementary material for: Integrating design-of-experiments (DOE) optimization and risk assessment towards a safe and simplified electroporation protocol for Toxoplasma gondii
Source: PLoS Negl Trop Dis. 2026 Apr 8;20(4):e0014194. doi: 10.1371/journal.pntd.0014194 (PMC13086436; doi:10.1371/journal.pntd.0014194)
Supplement: S1 Appendix — (DOCX) [file pntd.0014194.s005.docx]

# Validation of etScore to quantify *Toxoplasma* electroporation using fluorescence microscopy

Electroporation performance of mOM was compared to cytomix in 3 independent experiments. Briefly, 1x10^5^ tachyzoites were electroporated with 5 μg pTub:dYFP-pSAG1:CAT in complete cytomix or mOM as stated in the methods section of the main text and seeded onto HFF monolayers grown on Nunc Lab-Tek 8 chamber slides at 1x10^4^ tachyzoites per well. Infected cells were incubated for 48 h at 37 deg C/5% CO_2_. After incubation, cells were washed in DPBS to remove any non-invaded parasite, fixed with 4% PFA for 20 min at room temperature and blocked using 5% BSA/0.2% Triton X-100 in DPBS. Tachyzoites were labeled using a *Toxoplasma* rabbit polyclonal antibody (Thermofisher PA17252, 1:1000) and stained with anti-rabbit IgG-CFL 594 (Santa Cruz Biotechnology sc-516250, 1:500). Nuclei were counterstained with 1ug/ml Hoechst 33342. Slides were imaged using Olympus IX83 microscope at 20X and analyzed using Cellprofiler (1).

Presence of green parasites, expressing YFP was visually confirmed after the staining process (Fig S4A and B). Electroporation efficiency was calculated as a percentage of green parasites (YFP expression) over red parasites (antibody labeled) and parasite viability was calculated as a percentage of invaded parasites over number of host cells (percent parasitemia). Both quantities were standardized (equations 3 and 4) and combined as etScore (equation 5). Performance of both the buffers was also found to be similar using fluorescence microscopy-based assay (Fig S4C, D and E). This shows that our analysis method is not limited to datasets of different scales but can be applied when both metrics are percentage-based.

**References**

1. Stirling DR, Swain-Bowden MJ, Lucas AM, Carpenter AE, Cimini BA, Goodman A. CellProfiler 4: improvements in speed, utility and usability. BMC Bioinformatics. 2021 Sept 10;22:433.
