# Supplementary material for: Integrating design-of-experiments (DOE) optimization and risk assessment towards a safe and simplified electroporation protocol for Toxoplasma gondii
Source: PLoS Negl Trop Dis. 2026 Apr 8;20(4):e0014194. doi: 10.1371/journal.pntd.0014194 (PMC13086436; doi:10.1371/journal.pntd.0014194)
Supplement: S1 Table — (DOCX) [file pntd.0014194.s006.docx]

| **Primer ID** | **Sequence** |
| --- | --- |
| MUO-0087 | ATCCGGCCTTTATTCACATTCTTGCCCG |
| MUO-0088 | AACTGCCGGAAATCGTCGTGGTATT |
| MUO-0089 | GAGAGACGGGAACATCACTGGGCG |
| MUO-0090 | ACTGAAGGGGTTGGGAGAGAACACTT |
| MUO-0142 | GACCACGAAGCTCCCTTAACT |
| MUO-0143 | GAAACCGACATACAACCGTGTG |
| MUO-1016 | TTCTGTTTCGAGGGTCAACATG |
| MUO-1017 | CTATCGCTCTTCACCTTCTCCCTC |
