# Supplementary material for: Integrating design-of-experiments (DOE) optimization and risk assessment towards a safe and simplified electroporation protocol for Toxoplasma gondii
Source: PLoS Negl Trop Dis. 2026 Apr 8;20(4):e0014194. doi: 10.1371/journal.pntd.0014194 (PMC13086436; doi:10.1371/journal.pntd.0014194)
Supplement: S2 Table — (DOCX) [file pntd.0014194.s007.docx]

|  | Estimate | SE | t | P (>\|t\|) |  |
| --- | --- | --- | --- | --- | --- |
| Intercept | 1.171337 | 0.002330 | 502.827 | < 2x10^-16^ | *** |
| ATP | 0.336784 | 0.025782 | 13.063 | 2.72x10^-10^ | *** |
| EDTA | 0.216242 | 0.013055 | 16.564 | 6.35x10^-12^ | *** |
| GSH | 0.002331 | 0.040769 | 0.057 | 0.9551 |  |
| ATP:EDTA | -0.099064 | 0.035813 | -2.766 | 0.0132 | * |
| ATP:GSH | -0.113216 | 0.0049083 | -2.307 | 0.0339 | * |
| EDTA:GSH | -0.048814 | 0.043813 | -1.114 | 0.2807 |  |

Signif. codes: 0 ‘***’ 0.001 ‘**’ 0.01 ‘*’ 0.05 ‘.’ 0.1 ‘ ’ 1

Residual standard error: 0.5275 on 17 degrees of freedom; Multiple R-squared: 0.9892, Adjusted R-squared: 0.9854; F-statistic: 260.4 on 6 and 17 DF, p-value: 9.155x10^-16^
