# Supplementary material for: Integrating design-of-experiments (DOE) optimization and risk assessment towards a safe and simplified electroporation protocol for Toxoplasma gondii
Source: PLoS Negl Trop Dis. 2026 Apr 8;20(4):e0014194. doi: 10.1371/journal.pntd.0014194 (PMC13086436; doi:10.1371/journal.pntd.0014194)
Supplement: S3 Table — (DOCX) [file pntd.0014194.s008.docx]

|  | DF | SS | MS | F | P(>F) |  |
| --- | --- | --- | --- | --- | --- | --- |
| ATP | 1 | 253.952 | 253.952 | 912.6903 | 3.250x10^-16^ | *** |
| EDTA | 1 | 173.417 | 173.417 | 623.2518 | 7.769x10^-15^ | *** |
| GSH | 1 | 4.599 | 4.599 | 16.5302 | 0.0008042 | *** |
| ATP:EDTA | 1 | 0.908 | 0.908 | 3.2651 | 0.0885016 | . |
| ATP:GSH | 1 | 1.444 | 1.444 | 5.1895 | 0.0359249 | * |
| EDTA:GSH | 1 | 0.345 | 0.345 | 1.2413 | 0.2807236 |  |
| Residuals | 17 | 4.730 | 0.278 |  |  |  |

Significance codes: 0 ’***’ 0.001 ’**’ 0.01 ’*’ 0.05 ’.’ 0.1 ’ ’ 1
