# Supplementary material for: Integrating design-of-experiments (DOE) optimization and risk assessment towards a safe and simplified electroporation protocol for Toxoplasma gondii
Source: PLoS Negl Trop Dis. 2026 Apr 8;20(4):e0014194. doi: 10.1371/journal.pntd.0014194 (PMC13086436; doi:10.1371/journal.pntd.0014194)
Supplement: S4 Table — (DOCX) [file pntd.0014194.s009.docx]

|  | Estimate | SE | t | P(\|t\|) |  |
| --- | --- | --- | --- | --- | --- |
| Intercept | 9.8329x10^-1^ | 2.7577x10^-2^ | 35.6558 | < 2.2x10^-16^ | *** |
| ATP | 8.4809x10^-2^ | 1.0373x10^-2^ | 8.1763 | 4.103x10^-8^ | *** |
| EDTA | 2.6203x10^-1^ | 5.2393x10^-2^ | 5.0012 | 5.254x10^-5^ | *** |
| ATP^2^ | -1.0649x10^-2^ | 1.8138x10^-3^ | -5.8709 | 6.591x10^-6^ | *** |
| EDTA^2^ | -4.1422x10^-2^ | 3.2558x10^-2^ | -1.2722 | 0.2166 |  |
| ATP:EDTA | -1.3643x10^-2^ | 1.0184x10^-3^ | -13.3958 | 4.673x10^-12^ | *** |
| ATP^3^ | 5.0490x10^-4^ | 9.0265x10^-5^ | 5.5935 | 1.268x10^-5^ | *** |
| EDTA^3^ | 3.2938x10^-3^ | 5.3612x10^-3^ | 0.6144 | 0.5453 |  |

Significance codes: 0 ’***’ 0.001 ’**’ 0.01 ’*’ 0.05 ’.’ 0.1 ’ ’ 1

Multiple R-squared: 0.9847, Adjusted R-squared: 0.9798; F-statistic: 201.8 on 7 and 22 DF, p-value: < 2.2x10^-16^
