# Supplementary material for: Integrating design-of-experiments (DOE) optimization and risk assessment towards a safe and simplified electroporation protocol for Toxoplasma gondii
Source: PLoS Negl Trop Dis. 2026 Apr 8;20(4):e0014194. doi: 10.1371/journal.pntd.0014194 (PMC13086436; doi:10.1371/journal.pntd.0014194)
Supplement: S5 Table — (DOCX) [file pntd.0014194.s010.docx]

|  | DF | SS | MS | F | P(>F) |  |
| --- | --- | --- | --- | --- | --- | --- |
| FO(ATP, EDTA) | 2 | 296.28 | 148.14 | 301.7225 | < 2.2x10^-16^ | *** |
| PQ(ATP, EDTA) | 2 | 21.16 | 10.58 | 21.5509 | 6.563x10^-6^ | *** |
| TWI(ATP, EDTA) | 1 | 358.15 | 358.15 | 729.4463 | < 2.2x10^-16^ | *** |
| ATP^3^ | 1 | 17.63 | 17.63 | 35.9173 | 4.952x10^-6^ | *** |
| EDTA^3^ | 1 | 0.19 | 0.19 | 0.3775 | 0.5453 |  |
| Residuals | 22 | 10.80 | 0.49 |  |  |  |
| Lack of fit | 1 | 0.61 | 0.61 | 1.2491 | 0.2764 |  |
| Pure error | 21 | 10.20 | 0.49 |  |  |  |

Significance codes: 0 ’***’ 0.001 ’**’ 0.01 ’*’ 0.05 ’.’ 0.1 ’ ’ 1

FO: First Order; PQ: Pure Quadratic; TWI: Two Way Interactions
