# Supplementary material for: Integrating design-of-experiments (DOE) optimization and risk assessment towards a safe and simplified electroporation protocol for Toxoplasma gondii
Source: PLoS Negl Trop Dis. 2026 Apr 8;20(4):e0014194. doi: 10.1371/journal.pntd.0014194 (PMC13086436; doi:10.1371/journal.pntd.0014194)
Supplement: S6 Table — (DOCX) [file pntd.0014194.s011.docx]

**ANOVA table for Pure Quadratic model**

|  | DF | SS | MS | F | P(>F) |  |
| --- | --- | --- | --- | --- | --- | --- |
| FO(ATP, EDTA) | 2 | 296.28 | 148.14 | 124.2194 | 2.184x10^-13^ | *** |
| TWI(ATP, EDTA) | 2 | 369.91 | 369.91 | 310.1740 | 3.141x10^-15^ | *** |
| PQ(ATP, EDTA) | 1 | 9.40 | 4.70 | 3.9423 | 0.03308 | * |
| Residuals | 24 | 28.62 | 1.19 |  |  |  |
| Lack of fit | 3 | 18.43 | 6.14 | 12.6516 | 6.101x10^-05^ |  |
| Pure error | 21 | 10.20 | 0.49 |  |  |  |

Significance codes: 0 ’***’ 0.001 ’**’ 0.01 ’*’ 0.05 ’.’ 0.1 ’ ’ 1

Multiple R-squared: 0.9594, Adjusted R-squared: 0.9509

F-statistic: 113.3 on 5 and 24 DF, p-value: 6.958x10^-16^

FO: First Order; PQ: Pure Quadratic; TWI: Two Way Interactions

**ANOVA table for Full Cubic model**

|  | DF | SS | MS | F | P(>F) |  |
| --- | --- | --- | --- | --- | --- | --- |
| FO(ATP, EDTA) | 2 | 296.284 | 148 | 305.138 | 3.027x10^-16^ | *** |
| PQ(ATP, EDTA) | 2 | 21.162 | 11 | 21.795 | 7.526x10^-06^ | *** |
| I(ATP^3^) | 1 | 17.325 | 17 | 35.686 | 6.272x10^-06^ | *** |
| I(EDTA^3^) | 1 | 270.537 | 271 | 557.245 | < 2.2x10^-16^ | *** |
| TWI(I(ATP^2^), I(EDTA^2^)) | 1 | 87.184 | 87 | 179.578 | 9.264x10^-12^ | *** |
| TWI(ATP, EDTA) | 1 | 1.529 | 2 | 3.150 | 0.09043 |  |
| Residuals | 21 | 10.195 | 0 |  |  |  |
| Lack of fit | 0 | 0.000 | -Inf |  |  |  |
| Pure error | 21 | 10.195 | 0 |  |  |  |

Significance codes: 0 ’***’ 0.001 ’**’ 0.01 ’*’ 0.05 ’.’ 0.1 ’ ’ 1

Multiple R-squared: 0.9855, Adjusted R-squared: 0.98

F-statistic: 178.7 on 8 and 21 DF, p-value: < 2.2e-16

FO: First Order; PQ: Pure Quadratic; TWI: Two Way Interactions
