# Supplementary material for: Integrating design-of-experiments (DOE) optimization and risk assessment towards a safe and simplified electroporation protocol for Toxoplasma gondii
Source: PLoS Negl Trop Dis. 2026 Apr 8;20(4):e0014194. doi: 10.1371/journal.pntd.0014194 (PMC13086436; doi:10.1371/journal.pntd.0014194)
Supplement: S7 Table — (DOCX) [file pntd.0014194.s012.docx]

**Akaike Information Criterion (AIC)**

|  | DF | AIC |
| --- | --- | --- |
| Quadratic Model | 7 | -101.5051 |
| Balanced Model | 9 | -142.1228 |
| Full Cubic Model | 10 | -143.9419 |

**Bayesian Information Criterion (BIC)**

|  | DF | AIC |
| --- | --- | --- |
| Quadratic Model | 7 | -91.93407 |
| Balanced Model | 9 | -129.81710 |
| Full Cubic Model | 10 | -130.26890 |

**Likelihood Ratio Test (LRT)**

|  | Res. Df | RSS | DF | Sum of Sq | Pr (>Chi) |  |
| --- | --- | --- | --- | --- | --- | --- |
| Quadratic Model | 23 | 22.6316 |  |  |  |  |
| Balanced Model | 21 | 4.8589 | 2 | 17.7727 | < 2x10^-16^ | *** |
| Full Cubic Model | 20 | 4.2593 | 1 | 0.5995 | 0.09338 |  |

Significance codes: 0 ‘***’ 0.001 ‘**’ 0.01 ‘*’ 0.05 ‘.’ 0.1 ‘ ’ 1
