# Supplementary material for: Integrating design-of-experiments (DOE) optimization and risk assessment towards a safe and simplified electroporation protocol for Toxoplasma gondii
Source: PLoS Negl Trop Dis. 2026 Apr 8;20(4):e0014194. doi: 10.1371/journal.pntd.0014194 (PMC13086436; doi:10.1371/journal.pntd.0014194)
Supplement: S8 Table — (DOCX) [file pntd.0014194.s013.docx]

**Popular electroporation protocols for *Toxoplasma* transfection.**

| Device/Platform | Cell Count | DNA Amount (nmoles) | Cuvette (cm) | Voltage (KV) | Capacitance (µF) | Reference |
| --- | --- | --- | --- | --- | --- | --- |
| BTX ECM 630 | 10^7^ | 15-20 | 0.4 | 2.0 | N/A | [1] |
| BTX ECM 630 | 10^7^ | 3-5 | 0.1 | 0.5 | N/A | [1] |
| BTX Square Porator | 10^7^ | 55 | 0.4 | 1.7 | N/A | [2,3] |
| Bio-Rad Gene Pulser Xcell | N/A | N/A | 0.4 | 2.4 | 25 | [4] |
| Lonza 4D-Nucleofector | 10^7^ | N/A | N/A | Proprietary | Proprietary | [5] |
| General (N/A) | 10^7^ | N/A | 0.4 | 2.0 | 25 | [6] |
| Lonza Nucleofector 2b (Amaxa) | 10^6^-10^7^ | N/A | N/A | Proprietary | Proprietary | [7–9] |
| Our Protocol | 10^5^ | 5 | 0.4 | 1.8 | 3 | This study |

**References**

1. Upadhya R, Kim K, Hogue-Angeletti R, Weiss LM. Improved Techniques for Endogenous Epitope Tagging and Gene Deletion in Toxoplasma gondii. J Microbiol Methods. 2011;85: 103–113. doi:10.1016/j.mimet.2011.02.001

2. Sidik SM, Hackett CG, Tran F, Westwood NJ, Lourido S. Efficient Genome Engineering of Toxoplasma gondii Using CRISPR/Cas9. PLOS ONE. 2014;9: e100450. doi:10.1371/journal.pone.0100450

3. Markus BM, Bell GW, Lorenzi HA, Lourido S. Optimizing Systems for Cas9 Expression in Toxoplasma gondii. mSphere. 2019;4: 10.1128/msphere.00386-19. doi:10.1128/msphere.00386-19

4. Thaprawat P, Wang F, Chalasani S, Schultz TL, Di Cristina M, Carruthers VB. Toxoplasma gondii PROP1 is critical for autophagy and parasite viability during chronic infection. mSphere. 2025;10: e00829-24. doi:10.1128/msphere.00829-24

5. Bando H, Murata Y, Han Y, Sugi T, Fukuda Y, Bzik DJ, et al. Toxoplasma gondii chitinase-like protein TgCLP1 regulates the parasite cyst burden. Front Cell Infect Microbiol. 2024;14: 1359888. doi:10.3389/fcimb.2024.1359888

6. Albuquerque-Wendt A, Jacot D, Dos Santos Pacheco N, Seegers C, Zarnovican P, Buettner FFR, et al. C-Mannosylation of Toxoplasma gondii proteins promotes attachment to host cells and parasite virulence. J Biol Chem. 2020;295: 1066–1076. doi:10.1074/jbc.RA119.010590

7. Sugi T, Kato K, Weiss LM. An improved method for introducing site-directed point mutation into the Toxoplasma gondii genome using CRISPR/Cas9. Parasitol Int. 2016;65: 558–562. doi:10.1016/j.parint.2016.05.002

8. Roumégous C, Abou Hammoud A, Fuster D, Dupuy J-W, Blancard C, Salin B, et al. Identification of new components of the basal pole of Toxoplasma gondii provides novel insights into its molecular organization and functions. Front Cell Infect Microbiol. 2022;12. doi:10.3389/fcimb.2022.1010038

9. Wang P, Li S, Zhao Y, Zhang B, Li Y, Liu S, et al. The GRA15 protein from Toxoplasma gondii enhances host defense responses by activating the interferon stimulator STING. J Biol Chem. 2019;294: 16494–16508. doi:10.1074/jbc.RA119.009172
