# Supplementary material for: Integrating design-of-experiments (DOE) optimization and risk assessment towards a safe and simplified electroporation protocol for Toxoplasma gondii
Source: PLoS Negl Trop Dis. 2026 Apr 8;20(4):e0014194. doi: 10.1371/journal.pntd.0014194 (PMC13086436; doi:10.1371/journal.pntd.0014194)
Supplement: Attachment — Submitted filename: Dear Authors.docx [file pntd.0014194.s015.docx]

Dear Authors:

This manuscript proposes a simplified electroporation buffer (mOM) for T. gondii, optimized through DOE-based modeling and combined with a bowtie-style risk analysis. The topic is relevant and potentially impactful, especially for resource-limited laboratories. The inclusion of a biosafety framework is commendable. Nevertheless, several sections require clarification, additional data, and improved focus.

**Major Comments**

- The introduction clearly presents the need to simplify electroporation, but it lacks a detailed comparison with previously optimized buffers or electroporation media used for *T. gondii* (e.g., cytomix derivatives, commercial alternatives). Please better justify how mOM differs fundamentally rather than marginally from cytomix.
- While the design-of-experiments approach is valuable, details on replication, validation, and residual analyses are insufficient. Please clarify:
  - Number of replicates per design point.
  - Whether model assumptions (normality, homoscedasticity) were tested.
  - Justification for inclusion of cubic terms in the response-surface model.
  - Provide R² and adjusted R² values for all models.
- The “etScore” is a novel but somewhat arbitrary composite metric. Please validate it against conventional measures (e.g., flow cytometry-based transfection efficiency or fluorescence microscopy quantification). Without such comparison, reproducibility across different detection platforms remains uncertain.
- The manuscript often states “data from 4 experiments,” but it is unclear whether these represent independent biological replicates or technical repeats. Explicitly define both and adjust the statistical interpretation accordingly.
- The inclusion of a bowtie-based analysis is excellent, yet it dominates the Results section. Consider moving detailed descriptions to Supplementary Materials and summarizing key findings in a concise figure and table.
- Specify whether the assessment was performed following institutional biosafety standards (e.g., Canadian Biosafety Standards, WHO LSM 4th ed.), and indicate the biosafety level applied to *T. gondii* handling.
- Claims about applicability to “other apicomplexans” and “resource-limited settings” are somewhat speculative without experimental support. Please moderate these statements or add evidence of preliminary applicability.
- Figures 1–4 are described well but lack clarity on statistical dispersion (e.g., SD vs SEM).
- Include representative microscopy images showing typical electroporation outcomes (fluorescence expression, viability).

**Minor Comments**

- Ensure all references follow PLOS NTDs citation format and include DOI links.
- Add catalog numbers for all reagents where possible.
- The acknowledgments should clarify that biosafety evaluation did not involve live virulent strains.
- Provide details of ethical clearance (if applicable) for *T. gondii* strain use.

Below are detailed linguistic and stylistic comments organized according to the major sections of the manuscript.

### **1. Abstract**

**Main issues:**

- Sentences are too long and contain redundant phrases such as “Till date,” “This introduces complexity,” etc.
- Some statements are descriptive rather than result-focused.
- The abstract reads more like an introduction; it should emphasize **quantitative findings** and **key outcomes**.

### **2. Introduction**

**Main issues:**

- Excellent historical overview, but it is **too detailed and lengthy**.
- Paragraph transitions are weak, and the main research gap (complexity and variability of cytomix buffer) is not highlighted early.
- Frequent tense shifts (present ↔ past) and repetitive linking phrases.

### **3. Materials and Methods**

**Main issues:**

- Overly detailed reagent lists and repetition between tables and text.
- Verb tense inconsistency (switching between past and imperative).
- Some methods are written like instructions (e.g., “We suggest against using sharp needles…”), which is stylistically informal.

### **4. Results**

**Main issues:**

- Results are presented clearly but with heavy repetition and over-explanation of equations.
- The flow of logic is occasionally disrupted by detailed formula definitions that could be moved to Methods.
- Frequent use of “It was observed that…” slows down readability.
- Some subheadings are too long.

### **5. Discussion**

**Main issues:**

- Strong and comprehensive, but overly dense.
- Repetitive restatement of results rather than interpretation.
- Long sentences (30+ words) reduce readability.
- Some claims about applicability to “other apicomplexans” or “global accessibility” are speculative.

### **6. Conclusion**

**Main issues:**

- The section is strong in content but verbose and somewhat repetitive.
- Contains multiple speculative statements beyond the presented data.
- Some long sentences could be split for clarity.
