# Supplementary material for: Integrating design-of-experiments (DOE) optimization and risk assessment towards a safe and simplified electroporation protocol for Toxoplasma gondii
Source: PLoS Negl Trop Dis. 2026 Apr 8;20(4):e0014194. doi: 10.1371/journal.pntd.0014194 (PMC13086436; doi:10.1371/journal.pntd.0014194)
Supplement: Attachment — Submitted filename: 18022026response_to_reviewers.docx [file pntd.0014194.s017.docx]

PNTD-D-25-01842
Integrating Design-of-Experiments (DOE) Optimization and Risk Assessment Towards a Safe and Simplified Electroporation Protocol for Toxoplasma gondii
PLOS Neglected Tropical Diseases

Dear Dr. Ungrin,

Thank you for submitting your manuscript to PLOS Neglected Tropical Diseases. After careful consideration, we feel that it has merit but does not fully meet PLOS Neglected Tropical Diseases's publication criteria as it currently stands. Therefore, we invite you to submit a revised version of the manuscript that addresses the points raised during the review process.

Please submit your revised manuscript within by Feb 01 2026 11:59PM. If you will need more time than this to complete your revisions, please reply to this message or contact the journal office at plosntds@plos.org. When you're ready to submit your revision, log on to https://www.editorialmanager.com/pntd/ and select the 'Submissions Needing Revision' folder to locate your manuscript file.

Please include the following items when submitting your revised manuscript:

* A rebuttal letter that responds to each point raised by the editor and reviewer(s). You should upload this letter as a separate file labeled 'Response to Reviewers'. This file does not need to include responses to any formatting updates and technical items listed in the 'Journal Requirements' section below.
* A marked-up copy of your manuscript that highlights changes made to the original version. You should upload this as a separate file labeled 'Revised Manuscript with Track Changes'.
* An unmarked version of your revised paper without tracked changes. You should upload this as a separate file labeled 'Manuscript'.

If you would like to make changes to your financial disclosure, competing interests statement, or data availability statement, please make these updates within the submission form at the time of resubmission. Guidelines for resubmitting your figure files are available below the reviewer comments at the end of this letter.

We look forward to receiving your revised manuscript.

Kind regards,

Masoud Foroutan, Ph.D.
Academic Editor
PLOS Neglected Tropical Diseases

Susan Madison-Antenucci
Section Editor
PLOS Neglected Tropical Diseases

Shaden Kamhawi
co-Editor-in-Chief
PLOS Neglected Tropical Diseases
orcid.org/0000-0003-4304-636XX

Paul Brindley
co-Editor-in-Chief
PLOS Neglected Tropical Diseases
orcid.org/0000-0003-1765-0002

Journal Requirements:

1) Please ensure that the CRediT author contributions listed for every co-author are completed accurately and in full.
At this stage, the following Authors/Authors require contributions: Pratik Narain Srivastava, Nicholas Perewernycky, Lucca Filippo, Lianne M. Lefsrud, and Mark Ungrin. Please ensure that the full contributions of each author are acknowledged in the "Add/Edit/Remove Authors" section of our submission form.
The list of CRediT author contributions may be found here: <https://journals.plos.org/plosntds/s/authorship#loc-author-contributions>

**Response:** CRediT author contributions have now been updated.

2) We ask that a manuscript source file is provided at Revision. Please upload your manuscript file as a .doc, .docx, .rtf or .tex. If you are providing a .tex file, please upload it under the item type u2018LaTeX Source Fileu2019 and leave your .pdf version as the item type u2018Manuscriptu2019.

**Response:** Provided

3) We do not publish any copyright or trademark symbols that usually accompany proprietary names, eg ©,  ®, or TM  (e.g. next to drug or reagent names). Therefore please remove all instances of trademark/copyright symbols throughout the text, including:
- ® on page: 24.
- TM on pages: 2, 5, 23, and 24.

**Response:** All symbols have been removed.

4) Some material included in your submission may be copyrighted. According to PLOSu2019s copyright policy, authors who use figures or other material (e.g., graphics, clipart, maps) from another author or copyright holder must demonstrate or obtain permission to publish this material under the Creative Commons Attribution 4.0 International (CC BY 4.0) License used by PLOS journals. Please closely review the details of PLOSu2019s copyright requirements here: PLOS Licenses and Copyright. If you need to request permissions from a copyright holder, you may use PLOS's Copyright Content Permission form.
Please respond directly to this email and provide any known details concerning your material's license terms and permissions required for reuse, even if you have not yet obtained copyright permissions or are unsure of your material's copyright compatibility. Once you have responded and addressed all other outstanding technical requirements, you may resubmit your manuscript within Editorial Manager. 
Potential Copyright Issues:

- Figure S1.. Please confirm whether you drew the images / clip-art within the figure panels by hand. If you did not draw the images, please provide (a) a link to the source of the images or icons and their license / terms of use; or (b) written permission from the copyright holder to publish the images or icons under our CC BY 4.0 license. Alternatively, you may replace the images with open source alternatives. See these open source resources you may use to replace images / clip-art:
- https://commons.wikimedia.org
- https://openclipart.org/.

**Response:** The cell culture flask image used in Figure S1 is now changed to a hand drawn one.

5) Please amend your detailed Financial Disclosure statement. This is published with the article. It must therefore be completed in full sentences and contain the exact wording you wish to be published.
- State the initials, alongside each funding source, of each author to receive each grant. For example: "This work was supported by the National Institutes of Health (####### to AM; ###### to CJ) and the National Science Foundation (###### to AM)."
- State what role the funders took in the study. If the funders had no role in your study, please state: "The funders had no role in study design, data collection and analysis, decision to publish, or preparation of the manuscript.".
If you did not receive any funding for this study, please simply state: u201cThe authors received no specific funding for this work.u201d

**Response:** Funding statement has been updated accordingly.
 
Reviewers' Comments:

Reviewer's Responses to Questions

**Key Review Criteria Required for Acceptance?**


As you describe the new analyses required for acceptance, please consider the following:


**Methods**


-Are the objectives of the study clearly articulated with a clear testable hypothesis stated?

-Is the study design appropriate to address the stated objectives?

-Is the population clearly described and appropriate for the hypothesis being tested?

-Is the sample size sufficient to ensure adequate power to address the hypothesis being tested?

-Were correct statistical analysis used to support conclusions?

-Are there concerns about ethical or regulatory requirements being met?

Reviewer #1: (No Response)

Reviewer #2: (No Response)

Reviewer #3: (No Response)

**Results**


-Does the analysis presented match the analysis plan?

-Are the results clearly and completely presented?

-Are the figures (Tables, Images) of sufficient quality for clarity?

Reviewer #1: (No Response)

Reviewer #2: (No Response)

Reviewer #3: (No Response)

**Conclusions**


-Are the conclusions supported by the data presented?

-Are the limitations of analysis clearly described?

-Do the authors discuss how these data can be helpful to advance our understanding of the topic under study?

-Is public health relevance addressed?

Reviewer #1: (No Response)

Reviewer #2: (No Response)

Reviewer #3: (No Response)

**Editorial and Data Presentation Modifications?**


Use this section for editorial suggestions as well as relatively minor modifications of existing data that would enhance clarity. If the only modifications needed are minor and/or editorial, you may wish to recommend “Minor Revision” or “Accept”.

Reviewer #1: (No Response)

Reviewer #2: (No Response)

Reviewer #3: (No Response)

**Summary and General Comments**


Use this section to provide overall comments, discuss strengths/weaknesses of the study, novelty, significance, general execution and scholarship. You may also include additional comments for the author, including concerns about dual publication, research ethics, or publication ethics. If requesting major revision, please articulate the new experiments that are needed.

Reviewer #1:

Dear Authors:

This manuscript proposes a simplified electroporation buffer (mOM) for T. gondii, optimized through DOE-based modeling and combined with a bowtie-style risk analysis. The topic is relevant and potentially impactful, especially for resource-limited laboratories. The inclusion of a biosafety framework is commendable. Nevertheless, several sections require clarification, additional data, and improved focus.

Major Comments

- The introduction clearly presents the need to simplify electroporation, but it lacks a detailed comparison with previously optimized buffers or electroporation media used for T. gondii (e.g., cytomix derivatives, commercial alternatives). Please better justify how mOM differs fundamentally rather than marginally from cytomix.
  **Response:** Introduction has now been polished to make it concise and relevant details about other buffer systems have been included on page 4, lines 81 to 84
- While the design-of-experiments approach is valuable, details on replication, validation, and residual analyses are insufficient. Please clarify:
  - Number of replicates per design point.
    - This information has been inserted in lines 170, 205, 234, and 267 in respective figure legends.
  - Whether model assumptions (normality, homoscedasticity) were tested.
    - These assumptions were tested and the requested information has been inserted as weighted residual plots in the updated supplementary figure S2
  - Justification for inclusion of cubic terms in the response-surface model.
    - Justification for the inclusion of cubic terms has been inserted on page 12 at lines 224-232. Additionally, performance comparison of multiple tested models has also been added as supplementary tables S4-S7.
- Provide R² and adjusted R² values for all models.
- This information has been included in results at lines 228-235. Detailed comparison of all models has also been added in tables S5-S7.

The “etScore” is a novel but somewhat arbitrary composite metric. Please validate it against conventional measures (e.g., flow cytometry-based transfection efficiency or fluorescence microscopy quantification). Without such comparison, reproducibility across different detection platforms remains uncertain.

**Response:** The requested validation data has been collected and is presented in new Supplementary Appendix S1.

- The manuscript often states “data from 4 experiments,” but it is unclear whether these represent independent biological replicates or technical repeats. Explicitly define both and adjust the statistical interpretation accordingly.

**Response:** This statement has now been modified in the respective figure legends on pages 9, 11, 12, 14. Experimental and technical replicates are highlighted for each experiment. Any revised statistical interpretations (such as p-values) are modified accordingly.

- The inclusion of a bowtie-based analysis is excellent, yet it dominates the Results section. Consider moving detailed descriptions to Supplementary Materials and summarizing key findings in a concise figure and table.
  **Response:** We thank the reviewer for drawing this to our attention. We have edited the results section to be more concise and included concrete changes that we were able to incorporate in our workflow as a direct result of the systematic safety analysis. One of the reasons we included the bowtie in the main text is that such safety concerns are often viewed as an afterthought, and we wished to emphasize to the reader the importance of treating them as central to research in this area. While the risks encountered in this specific context are relatively minor, the rapid pace at which the ability to manipulate apicomplexans and other microorganisms is advancing means that there is potential for greater risk elsewhere. We therefore wish to use our work here to illustrate the availability of tools to assess and manage that risk. Consequently, we would prefer to retain this analysis in the main text, where it will have greater potential to influence standard practice.
- **Specify whether the assessment was performed following institutional biosafety standards (e.g., Canadian Biosafety Standards, WHO LSM 4th ed.), and indicate the biosafety level applied to *T. Gondii* handling.**
  **Response:** Biosafety approval for all material and protocols in this study are now stated in the methods section, page 21, lines 413-415.
- Claims about applicability to “other apicomplexans” and “resource-limited settings” are somewhat speculative without experimental support. Please moderate these statements or add evidence of preliminary applicability.
  **Response:** These changes have been made in the abstract.
- Figures 1–4 are described well but lack clarity on statistical dispersion (e.g., SD vs SEM).
  **Response:** Figure legends have been edited to provide this information.
- Include representative microscopy images showing typical electroporation outcomes (fluorescence expression, viability).
  **Response:** This information has been provided in Appendix S1.

Minor Comments

- Ensure all references follow PLOS NTDs citation format and include DOI links.

**Response:** References have been updated to match the PLOS NTD submission guidelines including DOI numbers.

- Add catalog numbers for all reagents where possible.

**Response:** Catalog numbers for all reagents are included in the relevant location.

- The acknowledgments should clarify that biosafety evaluation did not involve live virulent strains.
  **Response:** This information has been inserted on page 19, lines 387-391.
- Provide details of ethical clearance (if applicable) for T. gondii strain use.
  **Response:** Biosafety approval for all material and protocols in this study are now stated in the methods section, page 21, lines 413-415.

Below are detailed linguistic and stylistic comments organized according to the major sections of the manuscript.

1. Abstract

Main issues:

- Sentences are too long and contain redundant phrases such as “Till date,” “This introduces complexity,” etc.
- Some statements are descriptive rather than result-focused.
- The abstract reads more like an introduction; it should emphasize quantitative findings and key outcomes.

**Response:** Abstract has now been overhauled to make it more concise and avoid redundancy.

2. Introduction

Main issues:

- Excellent historical overview, but it is too detailed and lengthy.
- Paragraph transitions are weak, and the main research gap (complexity and variability of cytomix buffer) is not highlighted early.
- Frequent tense shifts (present ↔ past) and repetitive linking phrases.

**Response:** Introduction has now been polished and shortened.

3. Materials and Methods

Main issues:

- Overly detailed reagent lists and repetition between tables and text.
- Verb tense inconsistency (switching between past and imperative).
- Some methods are written like instructions (e.g., “We suggest against using sharp needles…”), which is stylistically informal.

**Response:** The section has now been polished and informal statements are removed.

4. Results

Main issues:

- Results are presented clearly but with heavy repetition and over-explanation of equations.
- The flow of logic is occasionally disrupted by detailed formula definitions that could be moved to Methods.
- Frequent use of “It was observed that…” slows down readability.
- Some subheadings are too long.

**Response:** Repeated phrases have now been moderated. Subheadings have also been changed to make them more suitable. Explanation of etScore has been streamlined on pages 6, 7 and 8.

5. Discussion

Main issues:

- Strong and comprehensive, but overly dense.
- Repetitive restatement of results rather than interpretation.
- Long sentences (30+ words) reduce readability.
- Some claims about applicability to “other apicomplexans” or “global accessibility” are speculative.

**Response:** These changes have been made in the discussion section on pages 18 and 19.

6. Conclusion

Main issues:

- The section is strong in content but verbose and somewhat repetitive.
- Contains multiple speculative statements beyond the presented data.
- Some long sentences could be split for clarity.

**Response:** The section has now been polished for clarity, difference from discussion and shorter sentences.

Reviewer #2: The manuscript presents a valuable and well-executed study that addresses a significant practical bottleneck in Toxoplasma gondii research: the complexity and variability of the standard electroporation buffer, cytomix. The development of a simplified, commercially available alternative (modified Opti-MEM, mOM) that performs equivalently with reduced variance is a noteworthy contribution. The integration of a novel scoring metric (etScore) and a formal risk assessment using bowtie analysis further strengthens the manuscript, making it of broad interest to the parasitology and molecular biology communities. The work is generally sound, but several major points require clarification and additional data to fully support the claims and ensure robustness.
Major Comments
- The manuscript effectively demonstrates that mOM works for a CRISPR/Cas9-mediated knockout at the TgUPRT locus. However, to establish mOM as a true replacement for cytomix, its performance should be validated across a wider range of common genetic manipulations. The authors should test mOM for:
- Transient Transfection: While RT-qPCR measures CAT mRNA, does this directly correlate with robust protein expression and fluorescence from a transiently transfected reporter (e.g., YFP) when assessed by microscopy or flow cytometry? A direct comparison of fluorescence levels and the percentage of YFP-positive parasites between cytomix and mOM would be highly convincing.
**Response:** We have included new experiments to compare mOM with cytomix for transient YFP expression using automated quantification and representative images as supplementary appendix S1.
- Stable Integration via Homologous Recombination: The TgUPRT knockout relies on non-homologous end joining (NHEJ) from a Cas9-induced break. It is crucial to test mOM with a standard stable transfection requiring homologous recombination (e.g., using a different locus or a traditional double-crossover strategy) to ensure it is broadly applicable.
**Response:** TgUPRT KO represented in this paper involves stable integration of pTub:dYFP-SAG:CAT in the UPRT locus leveraging a double-stranded break from a Cas9 targeting the UPRT locus (figure 5). Double crossover homologous recombination has been reported to be a very low frequency event in unmodified *Toxoplasma*. Most such studies use a KU80 knockout strain for further genetic modification, as it has diminished NHEJ capabilities, favoring integration but still requiring long flanking sequences (Fox et. al., Eukaryotic Cell 2009, 8(4), 520-9). Shen et. al. (2014) demonstrate that Cas9 assisted double-stranded break (DSB) does not need flanking sequences for integration, with the advantage that it does not require the KU80 knockout. We selected this strategy to demonstrate that mOM works with a Cas9 assisted DSB, achieving site-specific integration without any flanking sequences (“scarring”) in the UPRT locus (figure 5). While it would be interesting to confirm that mOM does not impact downstream DNA repair processes in the KU80 knockout strain, establishing that model for testing would go beyond the scope of the present study.
- The etScore is a central innovation. The description of its calculation, particularly the standardization step, needs more justification. Why was a min-max standardization chosen over Z-scoring? The authors state a constant of 1 was added to avoid division by zero, but this constant will disproportionately affect datasets with different ranges. The rationale for this specific mathematical construction should be elaborated.
**Response:** Additional description of mathematical rationales for using min-max standardization and addition of 1 as a constant are now included on page 7, lines 130-133. Equations 3 and 4 have also been modified to increase clarity.
- The claim that etScore is "easily adapted to different transgene detection methods" (e.g., fluorescence) is not demonstrated. The manuscript would be significantly strengthened by showing that etScore, calculated from flow cytometry data (e.g., %YFP+ cells for efficiency and a viability dye for cell health), yields similar conclusions regarding buffer performance. This is critical for labs that do not use RT-qPCR for routine efficiency checks.
**Response:** New experiments and data analysis have now been included as Supplementary Appendix S1 to demonstrate the applicability of etScore on fluorescence microscopy-based quantification, corroborating our RT-qPCR-based conclusions.

- The bowtie analysis is a commendable addition. However, its presentation feels somewhat disconnected from the specific protocol improvements made. The text should more explicitly link the risk analysis to the specific methodological changes adopted. For instance:
- The switch to a blunt needle for host cell shearing is an excellent, concrete outcome of the risk assessment. This should be highlighted as a direct mitigation for the "Percutaneous Inoculation" threat in the bowtie diagram and discussed prominently in the main text.
- Are there other specific protocol steps (e.g., avoiding aerosol-generating steps identified in the risk assessment) that were modified as a result of this analysis? Explicitly stating these would integrate the two halves of the paper more effectively.

**Response:** The requested changes have been made in the discussion section on pages 18 and 19.

- For the CCD optimization, the authors use a complex model with cubic terms. The justification for this over a standard quadratic model is that it reduced prediction errors, but the evidence for this (e.g., comparison of AIC/BIC or R² values between models) is not provided in the main text or supplementary tables (S4, S5). This justification should be clearly stated and the model diagnostics (Fig S3) should be briefly interpreted in the main text to bolster confidence in the model's validity.
**Response:** A comparison between a quadratic model, a balanced model (our model) and a cubic model are now included in the supplementary information tables S5, S6 and S7 using AIC/BIC and LRT. Discussion of model choice and validation has also been included in the main text.
- In Figure 1A, the authors use Levene's test to select 5 µg DNA based on homogeneity of variance, but then proceed with Welch's ANOVA, which does not assume equal variances. The logic here is circular. If variance homogeneity is the primary goal, stating that is fine, but the statistical narrative could be clearer.

**Response:** As an additional metric to show that 5 µg DNA group had the most consistent performance, coefficients of variance have now been included at lines 158 and 159 along with levene’s test for a clearer statistical narrative.

Minor Comments
1. Introduction:
- The introduction is comprehensive but could be more focused. Consider tightening the discussion on general transfection methods (biological, chemical) and placing greater emphasis on the specific history and documented drawbacks of cytomix in T. gondii research.
**Response:** The requested changes have been incorporated to make the introduction more concise.

2. Results:
- Figure 1: The Y-axis label in 1A and 1B should be "log2FC of CAT/TgGAPDH" for absolute clarity.

**Response:** Axis labels on figures 1A and 1B have been modified for clarity as requested.
- Section 3.2: When stating that GSH had no significant effect, it would be useful to report the p-value from the statistical model for the GSH factor to quantify the lack of effect.

**Response:** p-values from welch’s one way ANOVA are now included alongside the performance improvements in the results section.
- Section 3.4: The sentence "mOM yielding approximately a 2.7-fold reduction in variance" is clear, but the subsequent statement of a "63% reduction in variance between experiments" in the Discussion appears to be a different calculation (1 - (1/2.7) ≈ 0.63). Please use one consistent metric for clarity, preferably the fold-reduction.

**Response:** Percentage value has now been replaced with fold reduction.

3. Methods:
- Electroporation Parameters: The parameters (1.8 kV, 3 µF) are stated, but the resulting field strength (kV/cm) for the 0.4 cm cuvette should be calculated and mentioned, as this is a standard metric for comparing electroporation protocols.

**Response:** Field strength value has now been included in the methods section.
- RT-qPCR: It is stated that "Amplicons of approx. 180 bp were amplified". Please confirm the exact amplicon sizes for both CAT and TgGAPDH in the methods or a supplementary table.

**Response:** The exact amplicon sizes have now been mentioned in the methods section.
- Stable Transfection: For the stable knockout, the total DNA amount is 50 µg for 10^6 tachyzoites. This is a high DNA-to-parasite ratio. A comment on why this ratio was chosen, or a comparison to standard protocols using cytomix with this amount, would be helpful.

**Response:** This information has been inserted on page 23, lines 461 and 462.

4. Figures and Tables:
- Figure 3C/D: The contour plot (3D) is excellent. The 3D surface plot (3C) is less informative and could be moved to the supplement to save space.
- Figure 6 (Bowtie Diagram): This figure is dense and critical. In its current form, the font is very small and will be illegible in a published PDF. The authors must significantly improve the readability of this figure, potentially by simplifying the text or re-designing it across multiple panels.

**Response:** The bowtie diagram has now been made more concise.
- Table 5: The variants of mOM are clearly listed. It would be helpful to also include the final, optimized mOM buffer (OM + 2mM ATP + 3mM EDTA) in this table for a quick reference.

**Response:** The optimized version of mOM was found after the CCD experiment. Therefore, we are concerned that inclusion of these parameters in Table 5 would have potential to mislead the reader about the overall flow of the research.
Typos and Editorial Suggestions
• Abstract: "to across the international community" -> "to the international community"
• Introduction, Page 9, Line 85: "Crispr/Cas9" -> "CRISPR/Cas9"
• Results, Page 11, Line 113: "Table 1" is referenced, but the composition is described in the text on Page 10. Ensure table reference is correct.
• Results, Page 14, Line 190: "1, 2 and 5x10^5 tachyzoites" in the text, but the table header says "Tachyzoites (x10^5)" with values 1, 2, 5. This is inconsistent with the figure legend for 1B which says "1, 5 and 10x10^5". Please check and correct this discrepancy.
• Discussion: "scale-out experiments" – consider using "screening experiments" or "optimization experiments" for clarity.

**Response:** These points have been addressed

Reviewer #3:

This manuscript presents a well-executed and thoughtfully designed study that integrates Design-of-Experiments optimization with a detailed safety and risk-assessment framework to develop a simplified electroporation buffer (mOM) for Toxoplasma gondii. The work addresses a clear methodological bottleneck in Toxoplasma genetics: the complexity, batch-variability, and labor-intensive nature of the cytomix buffer. The authors’ use of RT-qPCR–based quantification, their introduction of the etScore metric, and the systematic DOE-based optimization of ATP and EDTA concentrations together represent a strong and innovative approach. The manuscript is generally well-written, methodologically rigorous, and the results are convincingly presented. The inclusion of a bowtie-based risk analysis is a notable strength that elevates the translational and practical value of the work.

Before acceptance, I suggest the authors clarify several methodological points to further strengthen transparency and reproducibility. For example, while the rationale for eliminating GSH is reasonable, the manuscript would benefit from a brief discussion comparing OM’s hypoxanthine levels with typical intracellular antioxidant conditions to support this interpretation.

**Response:** This information has now been clarified in the discussion section, page 18, lines 364-368.

The electroporation parameters are described clearly; however, a concise comparison with the most widely used protocols (e.g., those in the 2020 Methods in Molecular Biology volume) would help contextualize performance improvements.

**Response:** A table of popular electroporation protocols is now available in supplementary information table S8.

In the safety section, the bowtie diagrams are valuable but could be strengthened by specifying which controls the authors currently implement in their own laboratory versus which are recommended aspirationally for other settings. The conclusion would also benefit from a short statement on anticipated limitation, such as whether mOM performance may vary across Toxoplasma strains or CRISPR constructs.

**Response:** The bowtie diagram has been simplified in the revised manuscript. We have also mentioned the controls we identified and implemented in our protocol in the discussion section, page 19, lines 376 and 377. The statement regarding strain variation has also been included in the conclusion.

Overall, this is a strong and impactful manuscript that introduces a practical advancement likely to benefit many laboratories working with T. gondii and other apicomplexans. With minor clarifications and expansion of a few interpretive points, the manuscript will be suitable for publication.

**Response**: We thank the reviewer for their encouragement.

PLOS authors have the option to publish the peer review history of their article (what does this mean?). If published, this will include your full peer review and any attached files.


If you choose “no”, your identity will remain anonymous but your review may still be made public.


**Do you want your identity to be public for this peer review?** For information about this choice, including consent withdrawal, please see our Privacy Policy.

Reviewer #1: No

Reviewer #2: No

Reviewer #3: No

[NOTE: If reviewer comments were submitted as an attachment file, they will be attached to this email and accessible via the submission site. Please log into your account, locate the manuscript record, and check for the action link "View Attachments". If this link does not appear, there are no attachment files.]

Figure resubmission:

While revising your submission, we strongly recommend that you use PLOS’s NAAS tool (https://ngplosjournals.pagemajik.ai/artanalysis) to test your figure files. NAAS can convert your figure files to the TIFF file type and meet basic requirements (such as print size, resolution), or provide you with a report on issues that do not meet our requirements and that NAAS cannot fix.

After uploading your figures to PLOS’s NAAS tool - https://ngplosjournals.pagemajik.ai/artanalysis, NAAS will process the files provided and display the results in the "Uploaded Files" section of the page as the processing is complete. If the uploaded figures meet our requirements (or NAAS is able to fix the files to meet our requirements), the figure will be marked as "fixed" above. If NAAS is unable to fix the files, a red "failed" label will appear above. When NAAS has confirmed that the figure files meet our requirements, please download the file via the download option, and include these NAAS processed figure files when submitting your revised manuscript.

Reproducibility:

To enhance the reproducibility of your results, we recommend that authors of applicable studies deposit laboratory protocols in protocols.io, where a protocol can be assigned its own identifier (DOI) such that it can be cited independently in the future. Additionally, PLOS ONE offers an option to publish peer-reviewed clinical study protocols. Read more information on sharing protocols at https://plos.org/protocols?utm_medium=editorial-email&utm_source=authorletters&utm_campaign=protocols
